# Use of mHealth Apps by General and Family Medicine Physicians in Portugal: Observational Cross-Sectional Quantitative Study

**DOI:** 10.2196/88760

**Published:** 2026-09-30

**Authors:** Érica Taveira, Inês Dutra, Sílvia Rêgo

**Affiliations:** 1 Faculty of Medicine, University of Porto, Alameda Professor Hernâni Monteiro, Porto, 4200-319, Portugal, 351 22 040 2000; 2 Faculty of Sciences, University of Porto, Porto, Portugal; 3 R&D Department, Fraunhofer Portugal AICOS, Porto, Portugal; 4 RISE-Health, Faculty of Medicine, University of Porto, Porto, Portugal; 5 Comprehensive Health Research Centre (CHRC), Porto, Portugal

**Keywords:** mHealth apps, mobile health, general and family medicine, digital health adoption, mHealth

## Abstract

**Background:**

The digitalization of health care has accelerated the adoption of mobile health (mHealth) apps in general and family medicine in Portugal. These tools may support chronic disease management and clinical decision-making. However, limited high-quality scientific evidence and the absence of a national framework for certification and quality standards create uncertainty about their safe integration into clinical practice.

**Objective:**

This study aimed to characterize mHealth app use among general and family medicine residents and physicians in Portugal. It also examined factors influencing app selection, barriers to adoption, and clinicians’ perceptions regarding the integration of AI into clinical practice.

**Methods:**

An observational, cross-sectional, quantitative study was conducted using an online survey developed with LimeSurvey. The survey was distributed to residents and physicians registered with the *Ordem dos Médicos* (Portuguese Medical Association) who were in active clinical practice. Data were analyzed using descriptive statistics, with absolute and relative frequencies reported.

**Results:**

The final sample included 141 participants (n=104, 73.8% female; n=37, 26.2% male). Most clinicians were aware of mHealth apps (138/141, 97.9%), and 85.1% (120/141) reported using them in clinical practice. Among 120 users, 87.5% (n=105) regularly used 2 to 5 apps. A total of 69 unique apps were identified; the 13 most cited accounted for 77.4% (246/318) of all mentions, including Tonic, UpToDate, Cardio4all, and PEM Móvel. Apps were mainly used during clinical consultations (111/120, 92.5%). The most frequent factors influencing app choice were ease of use (114/120, 95.0%) and evidence-based clinical effectiveness (79/120, 65.8%). Reported barriers included lack of knowledge about available apps (117/139, 84.2%) and the absence of national evaluation standards (66/139, 47.5%). Among nonusers (n=21), the main structural barrier was poor integration with clinical information systems (n=15, 71.4%). Regarding AI, 56.0% (79/141) reported awareness of AI-integrated apps, mainly Tonic and ChatGPT. The same proportion considered AI use beneficial, especially for clinical decision support (114/141, 80.9%) and administrative automation (88/141, 62.4%). Key concerns included ethics, data security, and privacy (105/141, 74.5%) and limited interoperability (94/141, 66.7%).

**Conclusions:**

mHealth app adoption in Portugal is high but fragmented and largely driven by personal initiative (98/218, 45.0%) and informal recommendations, with limited institutional guidance. Tonic was the only app identified by respondents as reporting compliance with ISO 13485 (medical software quality), ISO/IEC 42001 (AI management systems), and UEMS-EACCME clinical accreditation. Most clinicians perceive national regulatory guidance as insufficient (73/139, 52.5%). Future progress requires the urgent development of a national framework for the curation and recommendation of mHealth apps aligned with international assessment frameworks such as DiGA (*Digitale Gesundheitsanwendungen* [Digital Health Applications], Germany) and DTAC (England), increased digital health training, and improved interoperability with clinical systems to ensure safe, effective, and equitable use in primary health care.

## Introduction

### Background

In Portugal, general and family medicine (GFM) is a core pillar of primary health care. GFM is defined as an “academic and scientific discipline—with its own educational content, research, evidence base, and clinical practice—and a clinical specialty focused on primary care” [1], according to the European consensus statement by the World Organization of Family Doctors (WONCA) Europe and the European Academy of Teachers in General Practice/Family Medicine (EURACT) [2].

In Portugal, the well-known title of “family physician” refers to a GFM specialist. In practice, GFM specialists deliver continuous, longitudinal care throughout the patient’s entire lifespan—spanning prevention, diagnosis, treatment, and rehabilitation—allowing for the development of a structured therapeutic relationship that integrates all biological, psychosocial, and cultural determinants into patient care [3-6]. GFM specialists function as part of multidisciplinary teams embedded in the community and coordinated with broader health care institutions [3].

In Portugal, health services are delivered across 3 main sectors—public, private, and social—with the public sector having the greatest predominance, mainly through the Portuguese National Health Service (*Sistema Nacional de Saúde* [SNS]) [3,4]. These 3 sectors operate with different organizational models, resources, and levels of technological integration. In addition, the digital transformation of health care has reconfigured clinical practices and assistive flows, culminating in opportunities and constraints that affect accessibility, continuity, and quality of health services [3,4].

Under the landmark 2024 structural reform, the public framework expanded the Family Health Unit model B (*Unidades de Saúde Familiar modelo B*) and established 39 Local Health Units (*Unidades Locais de Saúde*) to integrate primary care centers and hospitals under a unified management structure, aiming to maximize efficiency and continuity of care [7-9].

Conversely, the private sector is expanding rapidly, characterized by urban concentration, flexible operations, and a sharp rise in health insurance coverage—which grew from 14.0% in 2000 to 32% in 2021—driven primarily by barriers to accessibility in the public system and efforts to reduce wait times, despite persistent risks of care fragmentation [10,11].

Finally, the less-prominent social sector, comprising charitable organizations and private institutions of social solidarity, plays a crucial complementary role by providing community-based care to vulnerable populations, although its digital integration remains heavily constrained by financial limitations and uneven technological interoperability with the SNS [12,13].

Health systems are currently undergoing rapid digital transformation, which directly affects the clinical practice of GFM [14,15]. In this context, mobile health (mHealth) apps have emerged as strategic tools to support the management and monitoring of chronic diseases, assist clinical decision-making, and facilitate access to scientific evidence [14,16].

mHealth apps are key components of the rapid technological transformation currently underway in the health care sector. Within the conceptual framework of digital health, the World Health Organization (WHO) defines “eHealth” as the “cost-effectiveness and safe use of information and communication technologies (ICT) in support of health and health-related areas, including health services, health surveillance, health literature, health education, knowledge and research” [17]. Consequently, mHealth is an integral subset of eHealth, defined by WHO as “medical and public health practice through the use of wireless devices and technologies” [18].

According to Grundy [19], these apps can be classified into distinct categories, encompassing the management of chronic diseases, the monitoring of physiological parameters, health promotion and literacy, clinical practice support, and physician-patient communication. The potential and challenges of these technological tools in primary health care have already been evaluated across several countries. In Germany, Wangler and Jansky [20] conducted a written survey among primary care physicians to determine general perceptions regarding health applications, their experience with patient care, and the ideal conditions required to use these tools more effectively. In contrast, Mutebi and Devroey [21] developed a questionnaire-based survey in Belgium consisting of 2 distinct instruments—one for family physicians and another for the adult general population—to assess perceptions and mHealth app usage patterns in primary care provision.

In Australia, Byambasuren et al [22] supported the Royal Australian College of General Practitioners (RACGP) in its annual technology survey, deploying a web-based instrument to investigate knowledge of general practitioners (GPs), identify barriers and facilitators during consultations, and explore potential solutions to the identified obstacles. Similarly, in Hangzhou, China, Liu et al [23] carried out a questionnaire survey in community health centers to evaluate mobile device ownership and assess how and whether these professionals used applications for clinical decision-making or to obtain medical information.

Another highly relevant and contemporary focus in primary care is the integration of AI into clinical practice, either via mHealth platforms or standalone software. Consequently, this theme has been increasingly investigated using knowledge, attitudes, and practices frameworks to analyze the influencing factors, such as the cross-sectional online survey conducted by Chen et al [24] among Chinese physicians. In Denmark, Jørgensen et al [25] focused on identifying AI acceptance among GPs, specifically examining their perceptions of AI as a general concept to gather concrete insights and inform future strategic solutions within the Danish health care context. Furthermore, Martínez-Martínez et al [26] and Abdulazeem et al [27] gathered critical insights into the specific perceptions, barriers, and facilitators influencing AI adoption in primary care settings.

Finally, within the Portuguese context, Matias Mendes et al [28] conducted an online cross-sectional survey focused on distinguishing between Portuguese GPs who prescribe mHealth apps and those who do not, thereby successfully identifying the core factors that drive and sustain the clinical endorsement of these technological tools. However, despite this potential, there is an urgent need to raise awareness regarding the safe and appropriate use of properly certified mHealth apps, as well as to understand current perceptions of AI use in clinical practice in Portugal.

This landscape contrasts with established international assessment frameworks, such as DiGA (*Digitale Gesundheitsanwendungen* [Digital Health Applications]) in Germany [29] and DTAC in England [19,30]. Within these contexts, both patient-facing and physician-facing apps can be legally classified as medical devices, depending on their specific functionalities. Specifically, the German DiGA framework evaluates patient-facing, prescribable therapeutics, requiring digital applications to be formally classified as medical devices (under the European Union Medical Device Regulation [EU MDR 2017/745]) and to undergo rigorous evaluation by the Federal Institute for Drugs and Medical Devices (BfArM) to demonstrate their “medical benefit” and clinical performance. Similarly, England’s DTAC provides a broader framework that is highly relevant for physician-facing clinical decision support tools and includes stringent criteria for “clinical safety” to ensure that the algorithms and recommendations provided by these technologies are accurate, evidence-based, and secure for patient care.

Currently, regulations in force in Portugal establish that apps performing clinical functions (eg, diagnostics and monitoring of physiological parameters) should be classified as medical devices and require certification (subject to compliance with INFARMED and the EU MDR 2017/745) [31,32], leaving the majority of wellness and health literacy tools without certification.

Furthermore, even with robust digital infrastructure provided by Shared Services of the Ministry of Health of Portugal (*Serviços Partilhados do Ministério da Saúde* [SPMS]), the country lacks a formal evaluation model that systematically assesses clinical accuracy, safety, efficacy, and interoperability for these technological tools. This gap leads to an adoption pattern driven largely by individual initiative rather than systematic guidance [30,33-36]. Such unregulated use entails significant risks and unvalidated functionalities, including inaccurate or hallucinated recommendations. Clinical errors may arise from unvalidated monitoring or diagnostic functionalities [30,37-39], while the mass collection of sensitive data raises critical privacy concerns regarding General Data Protection Regulation (GDPR) compliance [40]. Furthermore, with the increasing integration of AI, there are growing and critical concerns regarding AI accuracy, the risk of algorithmic “hallucinations,” and the potential for the misinterpretation of results [41]. Operationally, the difficulties integrating these apps into existing clinical workflows can result in cognitive overload, while uneven adoption risks exacerbating digital inequality [39,42]. Therefore, establishing clear recommendations and guidelines, rigorous scientific validation, and continuous digital literacy programs (structured educational initiatives designed to provide essential technical and evaluative competencies) are vital to ensure the safety and effectiveness of these technologies in GFM [30,32].

### Objective

This study aimed to identify patterns of mHealth app use in GFM and explore factors influencing app selection, barriers to adoption, evidence gaps, and clinicians’ perspectives on AI integration into clinical practice. Given its exploratory and pilot nature, this study designed a baseline mapping to provide a comprehensive overview of the current digital landscape of mHealth apps in the context of GFM, considering that no similar research has been conducted in Portugal to date.

## Methods

### Study Design

This study used an observational, cross-sectional, quantitative design. Results were reported following the CHERRIES (Checklist for Reporting Results of Internet E-Surveys) checklist [43].

### Population and Sample

The target population included specialist physicians and residents in GFM with active clinical practice in Portugal. Eligibility required registration with the Portuguese Medical Association (*Ordem dos Médicos* [OM]) and current clinical practice in any health care sector (public, private, or social).

The study population was estimated based on the total number of GFM specialists registered with the Portuguese Medical Association as of May 19, 2024 (n=2393) [44], and the cumulative number of positions filled in the GFM program from 2020 to 2024 (n=9003) [45-49], resulting in a total population of 11,396 professionals. The minimum estimated sample size was 372, based on a population of 11,396 professionals, considering a 95% confidence level and a 5% margin of error.

Survey distribution had a nationwide scope, covering mainland Portugal and the Autonomous Regions of the Azores and Madeira. Participants were recruited using convenience sampling, supported by a snowball dissemination strategy. Specifically, formal invitations were emailed to the Portuguese Association of General and Family Medicine (*Associação Portuguesa de Medicina Geral e Familiar*), Local Health Units (*Unidades Locais de Saúde*), and charitable health care organizations (*Misericórdias*) from all over Portugal. Additionally, the study was promoted through a dedicated post on LinkedIn, a professional social media platform. This post detailed the study’s participation criteria and target population and provided a direct link to the survey. Simultaneously, the research team leveraged their professional networks to encourage peer-to-peer sharing.

### Instrument and Data Collection

Data were collected using an open online survey hosted on LimeSurvey (University of Porto) and active from May 26 to August 26, 2025. Prior to distribution, a pretest was conducted with 3 researchers in the field (not included in the main study) and 3 GFM specialists. This validation step allowed for the refinement of the survey, ensuring that items related to mHealth app use were clear, objective, and specifically tailored to the context of GFM practice, in addition to assessing completion time.

The survey was structured into 7 sections to cover the full scope of the research topic, with each item displayed on a separate page to enhance participant focus (Multimedia Appendix 1). Although the instrument comprised 193 items, the adaptive branching logic ensured that the actual number of questions varied according to the participant’s profile. The session commenced with the display of the informed consent form (Q1); participants who did not provide consent were automatically directed to the end of the survey. Those who agreed proceeded to the common core (Sections 1-3; Q2-Q15), where the minimum requirement was 12 mandatory questions, with a potential maximum of 14. These sections focused on the characterization of demographic data, practice patterns, and the use of mHealth apps in GFM (Sections 1‐3).

Following Section 3, the path diverged as mHealth app users transitioned to Section 4 (Q16-Q18; 2‐3 items) for the identification and characterization of mHealth apps, where they were further routed to one of 3 specific subsections based on their reported number of apps used: Section 4.1 (Usage of 1 mHealth app; Q19-Q36; 9‐18 items), Section 4.2 (Usage of 2‐5 mHealth apps; Q37-Q74; 12‐38 items), or Section 4.3 (Usage of 6‐10 mHealth apps; Q75-Q137; 12‐63 items).

Nonusers proceeded directly to Section 5 (Q138-Q170; 5‐33 items) to assess knowledge regarding the use of mHealth apps in the context of GFM. Following these conditional paths, participants from both groups converged at Section 6 (Q171-Q182; 10‐12 items), which addressed opinions and perspectives on currently available mHealth apps, and proceeded to Section 7 (Q183-Q193; 5‐11 items), which focused on the integration of AI into mHealth apps within GFM clinical practice.

Upon completion of the survey, a hyperlink was provided for participants who wanted to receive the study results. This hyperlink directed them to a separate and independent LimeSurvey survey (also hosted on the University of Porto servers), ensuring that email addresses would remain unlinked to the primary survey responses to maintain total anonymity of the participants.

The LimeSurvey (University of Porto) platform enforced real-time completeness checks, preventing participants from advancing to the next item if mandatory fields—clearly identified for participants and marked with an asterisk (*)—were not filled. The questionnaire used a mix of single-choice, multiple-choice, Likert scale, and open-ended questions. Where appropriate, an “Other” response option was provided, including an open-text field to allow participants to further elaborate on their choices if they wished to do so.

Item randomization was not performed. Additionally, a review step was included, providing a “back” button that allowed participants to review and modify their previous responses before final submission. To prioritize the user experience and ensure strict participant anonymity, tracking via persistent cookies and CAPTCHA verification was disabled. Additionally, due to privacy settings, external web traffic tracking tools were not used. Consequently, passive views of the informed consent page could not be recorded. The LimeSurvey platform counted unique visitors only after a session was initiated, using temporary session identifiers.

Access to the questionnaire was provided via direct URL distribution, and the study was excluded from the institutional platform’s public index. Given the reliance on intermediary institutions (gatekeepers) to disseminate the survey to the target population, the statistical analysis of participation was limited to calculating the completion and dropout rates, as detailed in the response ratios in the *Results* section.

### Ethical Considerations

The study protocol was reviewed and approved by the Ethics Committee of the Faculty of Medicine, University of Porto (reference: 322/CEFMUP/2025). All participants provided informed consent prior to participation. This consent was obtained through a detailed information sheet displayed on the first page of the digital platform before the survey commenced. This page detailed the study’s objectives, stipulated that participation was strictly voluntary, indicated that the survey required approximately 8 minutes to complete, and outlined the data collection process.

Data were collected anonymously and used solely for research purposes, in accordance with Portugal’s Data Protection Law (Law number 58/2019 of August 8) and the European GDPR (Regulation [EU] 2016/679) [40]. All responses were anonymized, treated with strict confidentiality, and securely stored on the University of Porto’s servers for a period of 5 years. Furthermore, participants were informed of their right to request study results from the research team. No financial or other incentives were provided to participants for their involvement in the study.

### Data Analysis

Descriptive statistics were used to summarize the data, including absolute and relative frequencies and proportions. Analyses were conducted using IBM SPSS, Microsoft Excel, and Python. For single-choice questions, percentages were calculated based on the total number of respondents, while for multiple-response questions, proportions were calculated relative to the total number of responses. Although the primary analysis included only complete and submitted questionnaires, a pairwise deletion strategy was applied to a single section (Section 6) where isolated item nonresponse occurred; consequently, percentages for those specific variables were computed based on the number of valid responses.

## Results

### Response Ratios

During the survey distribution period, a total of 323 unique survey sessions were initiated. Of these, 141 completed the entire questionnaire, yielding a completion rate of 43.7% (141/323). Conversely, 182 sessions were abandoned prior to final submission, resulting in a dropout rate of 56.3% (182/323), with the highest attrition observed in Section 4 of the questionnaire. Regarding response burden, the mean completion time among respondents was 16.6 (SD 18.7) minutes, with a median completion time of 13.0 (IQR 9.9-16.3) minutes.

### Sample Characteristics and Usage Patterns

Of the 141 participants, the majority were female participants (n=104, 73.8%). Regarding their professional profile, 100 (70.9%) were specialists in GFM, and 65 (46.1%) had between 5 and 15 years of clinical practice. The predominant age groups were 26 to 35 years (n=61, 43.3%) and 36 to 55 years (n=55, 39.0%). Concerning their workplace, most participants were employed in the public sector (n=98, 69.5%), predominantly practicing in the districts of Porto (n=38, 27.0%) and Lisboa (n=33, 23.4%), as shown in Table 1.

**Table 1.** Demographic characteristics of the sample (N=141).

Characteristic	Participants, n (%)
Age (y)	
<26	1 (0.7)
26‐35	61 (43.3)
36‐45	55 (39.0)
46‐55	16 (11.3)
56‐65	5 (3.5)
66‐75	3 (2.1)
Sex	
Female	104 (73.8)
Male	37 (26.2)
Function	
Specialist physicians	100 (70.9)
Residents	41 (29.1)
Years of clinical practice in general and family medicine	
<5	43 (30.5)
5‐15	65 (46.1)
≥16	33 (23.4)
Sector of clinical practice in general and family medicine	
Public	98 (69.5)
Public and private	33 (23.4)
Others	10 (7.1)
Districts where participants practice general and family medicine	
Porto	38 (27.0)
Lisboa	33 (23.4)
Aveiro	21 (14.9)
Braga	12 (8.5)
Setúbal	9 (6.4)
Faro	5 (3.5)
Others	23 (16.3)

The remaining districts where participants practice GFM are detailed in Table S1 in Multimedia Appendix 2.

Awareness of mHealth apps was nearly universal. Among 141 participants, 138 (97.9%) confirmed recognition of these technological tools. Moreover, the prevalence of use was high, with 120 (85.1%) participants reporting the active use or prescription of these tools (Table 2).

**Table 2.** Awareness and use of mobile health (mHealth) apps in the clinical practice of general and family medicine (N=141).

Response	Participants, n (%)
Awareness of mHealth apps	
Yes	138 (97.9)
No	3 (2.1)
Use and/or prescription of mHealth apps	
Yes	120 (85.1)
No	21 (14.9)

The primary benefits reported by 120 mHealth app users were access to clinical evidence (n=115, 95.8%) and digital checklists or guidelines (n=89, 74.2%), followed by automatic alerts for drug interactions and contraindications (n=43, 35.8%). In the open-ended question (Q13), several users highlighted the importance of using clinical calculators (*“*Use of calculators that assist in clinical practice and other scientific information” and “For calculating clinical scores that can guide my clinical practice*”*) and standard verification, emphasizing the role of these tools in point-of-care medical decisions.

Among the subgroup of 21 nonusers, participants evaluating hypothetical benefits highlighted access to clinical evidence (n=15, 71.4%), automatic alerts for drug interactions and contraindications (n=14, 66.7%), and the availability of real-time data (n=10, 47.6%). In the open-ended question (Q15), one comment reinforced this perception: “I suppose there are multiple potential benefits (probably all the listed options apply), but technology fatigue is brutal,” suggesting that digital overload may hinder adoption despite recognized value.

Table S2 in Multimedia Appendix 2 outlines the benefits perceived by mHealth app users and nonusers as hypothetical users. Among the subgroup of 120 mHealth app users, 87.5% (n=105) reported using between 2 and 5 apps regularly in clinical practice. The majority of these users reported daily use (n=69, 57.5%), followed by weekly use (n=46, 38.3%), monthly use (n=4, 3.3%), and annual use (n=1, 0.8%). Regarding the operating system of their primary mobile device, 58.3% (n=70) used Android, while 39.2% (n=47) used iOS. Additionally, 2.5% (n=3) specified using the public computer systems at health centers (Windows/Web) to access certain features, rather than relying exclusively on personal mobile devices.

In terms of the contexts in which mHealth apps were used, medical consultations were the most frequently selected context, reported by 111 (92.5%) app users. Other selected contexts included clinical planning or case review outside of consultations (n=73, 60.8%) and continuing medical education (n=57, 47.5%). Table 3 details the full breadth of contexts of mHealth app use selected by the participants.

**Table 3.** Contexts of use of mHealth apps (n=120).

Context of use	Participants^a^, n (%)^a^
During the medical consultation	111 (92.5)
Outside the consultation for clinical planning or case review	73 (60.8)
Continuous medical education	57 (47.5)
Urgency or emergency situations	34 (28.3)
Direct patient counseling	33 (27.5)
Communication with other professionals	32 (26.7)
Remote patient monitoring	6 (5.0)
Other	1 (0.8)

a Participants could select more than one response.

### Purposes and Most-Used Apps

The primary purposes of mHealth app use were utilitarian, focusing on immediate clinical decision support. Key functions included the use of calculators and clinical assessment tools (111/120, 92.5%), support for clinical decision-making (105/120, 87.5%), and the search for scientific evidence to support clinical practice (102/120, 85.0%). In addition, 3.3% (4/120) of participants reported other specific purposes, namely electronic prescribing, the use of large language models for medical purposes (eg, BioBERT, Med-PaLM, and Med-PaLM 2) for rapid evidence retrieval and updating clinical guidelines from various medical societies, and the transcription of printed medical complementary diagnostic and therapeutic test results. A comprehensive overview of all reported purposes of use is provided in Table 4.

**Table 4.** Purposes for using mHealth apps in the clinical practice of general and family medicine (n=120).

Purposes	Participants^a^, n (%)
Calculators and clinical assessment tools	111 (92.5)
Support for clinical decision-making	105 (87.5)
Searching for scientific evidence	102 (85.0)
Health education/awareness	52 (43.3)
Prescription as part of treatment plan	45 (37.5)
Communication with patients	20 (16.7)
Prescription for follow-up/monitoring	18 (15.0)
Sharing of anonymized clinical cases	17 (14.2)
Management of appointment/scheduling/records	15 (12.5)
Management/monitoring of medication adherence	14 (11.7)
Data sharing (institutional data flows)	13 (10.8)
Other purposes	4 (3.3)

a Participants could select more than one response.

A total of 69 different apps were identified, with the 13 most frequently mentioned accounting for 246 of the 318 (77.4%) responses. Among the 120 users, the top apps reported were Tonic (67/120, 55.8%), UpToDate (34/120, 28.3%), Cardio4all (26/120, 21.7%), PEM Móvel (26/120, 21.7%), MDCalc (25/120, 20.8%), easyPed (22/120, 18.3%), Medscape (13/120, 10.8%), ESC CVD Risk Calculator (9/120, 7.5%), and SNS 24 (8/120, 6.7%). Finally, ChatGPT, Elegibilidade THM, MGFamiliar, and Poupe na receita were each reported by 3.3% (4/120) of users. The remaining mHealth apps that were identified are listed in Table S3 in Multimedia Appendix 2.

### Selection Criteria and Barriers

Ease of use (114/120, 95.0%) emerged as the dominant criterion for app selection, followed by proven clinical efficacy (eg, randomized controlled trials or real-world evidence: 79/120, 65.8%)—which in this context serves as a fundamental proxy for the accuracy and safety of clinical recommendations—and cost of the app (64/120, 53.3%). Table S4 in Multimedia Appendix 2 outlines the remaining selection criteria.

In addition, in the evaluation of mHealth quality criteria, the most valued aspects were clinical relevance for GFM (102/120, 85.0%); efficacy validated by scientific studies—a key indicator of perceived diagnostic and therapeutic accuracy (77/120, 64.2%); and an intuitive and accessible interface (77/120, 64.2%). Table S5 in Multimedia Appendix 2 lists the remaining quality criteria.

As illustrated in Table 5, regarding the total number of responses (218 selections), users were largely self-motivated in their choice of apps (98/218, 45.0%) or influenced by colleagues (66/218, 30.3%), while the influence of the Directorate-General of Health (DGS) was minimal (3/218, 1.4%).

**Table 5.** Sources of recommendations for the use of mobile health (mHealth) apps (n=218).

Recommendations for the use of mHealth apps	Participants^a^, n (%)
By individual initiative	98 (45.0)
Recommendation by one or more colleagues	66 (30.3)
Recommendation by the Portuguese Association of General and Family Medicine	12 (5.5)
Other recommendations from medical specialties other than general and family medicine	9 (4.1)
Recommendation by another association, society, council (national or international) or from a medical specialty other than general and family medicine	8 (3.7)
Recommendation by the Directorate-General of Health (DGS)	7 (3.2)
Institutional recommendation from the workplace	5 (2.3)
Official guideline issued by the DGS	3 (1.4)
Official guideline issued by the World Health Organization (WHO)	3 (1.4)
Recommendation by the WHO	3 (1.4)
Official workplace guideline from the institution	2 (0.9)
Recommendation by the specialty board of general and family medicine	2 (0.9)

a Participants could select more than one response.

Among the subgroup of nonusers (n=21), structural reasons prevailed for the nonuse of mHealth apps. These included a lack of integration with existing clinical systems—with qualitative feedback specifically pointing to SClínico, the primary electronic health record system of the SNS—and insufficient time to explore new tools (15/21, 71.4% each). A lack of familiarity with these tools and a deficiency in technical support were also reported (9/21, 42.9% each). Table S6 in Multimedia Appendix 2 outlines the less prevalent reasons.

When considering the total valid sample for this barrier analysis (n=139), the primary barrier to adoption was a lack of awareness regarding existing apps (117/139, 84.2%), followed by the absence of national guidelines and endorsement by scientific societies (66/139, 47.5% each). The remaining barriers are presented in Table S7 in Multimedia Appendix 2.

### Perceptions About the Role of the SNS

Regarding the available scientific evidence for validating mHealth apps, most respondents considered it sufficient (82/139, 59.0%), while over one-third (45/139, 32.4%) rated it as insufficient or barely sufficient. High confidence in the quality and safety of the information provided by these applications predominated (62/139, 44.6%), followed by moderate confidence (54/139, 38.8%), while only 7.2% (10/139) reported low or very low confidence. Furthermore, a strong consensus emerged regarding the role of knowledge, with 80.6% (112/139) of participants agreeing that familiarity—or the lack thereof—influences application use in clinical practice, compared to only 5.8% (8/139) who disagreed.

In contrast, more than half of the respondents (73/139, 52.5%) disagreed that national guidelines and recommendations are sufficient to support primary care physicians in deciding whether to use these tools; 36.7% (51/139) remained neutral, and only 10.8% (15/139) agreed. Finally, strong support was observed for the integration of mHealth apps into the SNS, with 80.6% (112/139) in agreement, 15.8% (22/139) neutral, and only 3.6% (5/139) in disagreement. In conclusion, these Likert-scale responses indicate an overall positive attitude regarding trust and the future integration of mHealth into the SNS, while simultaneously revealing criticisms regarding the lack of scientific validation and gaps in national regulatory guidance.

The dichotomous-response questions reinforce this pattern. The vast majority of physicians stated that the DGS does not provide sufficient outreach and training (133/139, 95.7%), yet expressed strong interest in receiving additional training (120/139, 86.3%). This perceived training gap is accompanied by a clear expectation of institutional action, with 78.4% (109/139) arguing that the SNS should issue specific guidelines for mHealth use, explicitly demanding centralized information on available applications (104/139, 74.8%) and independent scientific evaluations (103/139, 74.1%). Taken together, these results underscore the need to strengthen capacity-building policies and formal regulation.

In line with the lack of a regulatory framework, the most frequently cited barrier was a lack of awareness regarding existing applications (117/139, 84.2%), followed by the absence of national guidelines or guidance from scientific societies (66/139, 47.5% each) and data privacy concerns (42/139, 30.2%), whereas user resistance was less prevalent (29/139, 20.9%). Open-ended responses reinforced these findings, highlighting several structural and operational barriers (Multimedia Appendix 2, Table S8).

### Perception of AI

More than half of the participants (79/141, 56.0%) were aware of apps integrating AI, with an equal proportion acknowledging its potential benefits for clinical practice. Regarding the 67 valid responses that specified known apps, the most prominent were TonicApp (n=27, 40.3%), ChatGPT (n=10, 14.9%), and OpenEvidence (n=8, 11.9%), alongside sporadic mentions of Medical AI, Med-PaLM, AMBOSS, Perplexity, and the SNS 24 app (n=1 each). Associated comments suggested a baseline conceptual awareness but limited technical specificity (eg, “I know they exist to transcribe consultations and create summaries”; “I don’t remember; I don’t use them”).

The perceived potential of AI was highest for clinical decision support (114/141, 80.9%) and administrative automation (88/141, 62.4%), while telemedicine or triage (36/141, 25.5%) and remote monitoring (35/141, 24.8%) were highlighted less frequently. One open-ended response illustrated the aspirational use of AI during consultations: “Being face-to-face with the patient while the app transcribes the conversation into organized text on the computer.”

Conversely, the primary reported challenges were ethical and privacy concerns (105/141, 74.5%), alongside training and interoperability deficits (94/141, 66.7% each). Algorithm reliability and the risk of error were feared by 54.6% (77/141), followed by acquisition costs (69/141, 48.9%) and resistance from health care professionals (61/141, 43.3%). Only 0.7% (1/141) did not identify any challenges, while a single respondent raised concerns regarding the environmental impact of AI infrastructure.

Regarding transparency, only 40.4% (57/141) of mHealth app users were actively aware that their tools incorporated AI functionalities; 5.7% (8/141) stated they did not, 31.2% (44/141) did not know, and 22.7% (32/141) did not answer. This significant lack of awareness, even among current users, points to low transparency in commercial software solutions and highlights a pressing need to strengthen digital AI literacy among medical professionals.

Perceptions regarding AI’s impact on the physician-patient relationship were highly divided: 37.6% (53/141) anticipated an improvement, 15.6% (22/141) foresaw no significant impact, 12.8% (18/141) feared potential harm or depersonalization, and 34.0% (48/141) expressed no opinion. Supplementary feedback indicated that optimism stems from AI’s ability to free up face-to-face time for the patient by reducing bureaucratic burdens and assisting with clinical documentation. However, uncertainty persists, and a cautious minority remains concerned about the dehumanization of care.

Spontaneous feedback perfectly reflected this baseline encapsulation of enthusiasm, caution, and skepticism all at once, clustering around several core themes and illustrative perspectives (Multimedia Appendix 2, Table S9).

## Discussion

### Principal Findings

The high adoption rate of mHealth apps in Portuguese GFM (120/141, 85.1%) is notable; however, their usage is informal and fragmented. Matias Mendes et al [28], whose research specifically focused on the prescription of mHealth apps in Portugal, identified a limited adoption rate (45.2%) driven largely by personal initiative rather than institutional strategy. Similarly, our findings suggest that the active prescription of apps to patients remains low; this is corroborated by the finding that the use of these technologies is driven primarily by personal initiative (98/218, 45.0% responses) or by trust in colleagues (66/218, 30.3% responses).

Portuguese physicians and residents prioritize ease of use (114/120, 95.0%) and proven clinical efficacy (79/120, 65.8%). Although the specific term “accuracy” was not a standalone option in the survey, it is deeply embedded in these findings, as physicians clearly demand scientific validation to ensure the accuracy of the tools they use. This aligns with the technology acceptance model [50]. Nevertheless, this emphasis on pragmatic factors over security and privacy (27/120, 22.5%) and regulatory compliance (≤20%) indicates a mismatch with the requirements of the EU MDR [32] and the GDPR [40]. Additionally, the relatively “low” percentage (66/139, 47.5%) of physicians viewing the absence of national guidelines as a barrier reflects, once again, a culture of “clinical pragmatism” sustained by a historical regulatory vacuum in Portugal, where physicians have normalized operating without official endorsement. However, this contrasts with the opinion of physicians from other European countries, such as Germany and Belgium, who state that they do not use mHealth in their clinical practice due to concerns about data privacy, security, and legal liabilities [20,21].

Overall, the participant sample was younger than the physician population registered with the Portuguese Medical Association (OM) during the study period [44]. This divergence occurred because the ages of our participants were primarily concentrated in the 26 to 35 years (61/141, 43.3%) and 36 to 55 years (55/141, 39.0%) age groups, whereas 45.0% (n=4115) of the physicians registered with the OM were over 65 years of age and more than 18.0% were over 70 years of age.

However, the sample aligns well with national data in terms of sex and geographic distribution; females represented 73.8% (104/141) of our sample compared to 64.3% (n=5788) reported by the OM. Regarding geography, the majority of our participants worked in Porto (38/141, 27.0%) and Lisbon (33/141, 23.4%), which mirrors the notable concentrations registered by the OM in the districts of Porto (21.4%) and Lisbon (21.6%) [44].

Given that the study was disseminated via email and social media, the web-based survey format naturally attracted younger, more tech-savvy participants with a baseline interest in and knowledge of the topic. Consequently, this study is not fully representative of the broader national population of GFM physicians, a limitation further compounded by the fact that the target sample size was not achieved (141 participants obtained instead of the calculated sample size of 372). Furthermore, the subgroup of mHealth app users (n=120) is highly disproportionate compared to the subgroup of nonusers (n=21).

Motivation and digital literacy significantly influence recruitment in digital health research. Matias Mendes et al [28] observed a median age of 36 (IQR 31.8-43.0) years among Portuguese physicians, aligning closely with the demographic profile of our study. Those authors highlighted the presence of selection biases, noting that the online distribution of surveys disproportionately attracts clinicians with high digital engagement. Correspondingly, the study by Mutebi and Devroey [21] in Belgium mirrors our dominant age cohort, reporting that 91% of surveyed family physicians fell within the 26 to 35 years age bracket. Such difficulties in obtaining representative samples and the impact of “survey fatigue” are well-documented, as evidenced by low response rates in a study conducted in Australia (4.6%) and limited sampling in Denmark (109 responses, of which 92 were complete) [22,25].

The strong concentration of our sample in the 26 to 35 and 36 to 55 years age groups (which together account for more than 82% of participants) is key to understanding the extremely high rate of informal adoption (85.1%) observed. Age may be associated with the use of these technologies. In a study by Wagner and Jansky [20] in Germany, physicians younger than the average age view these applications much more favorably than their older colleagues (46% vs 23%). In Australia, a study by Byambasuren et al [22] found that the frequency of app recommendations drops sharply as the number of years of clinical practice increases.

The main documented exception is found in the study by Chen et al [24] in China regarding the topic of AI, where senior physicians (ages 50‐59) demonstrated greater knowledge—a phenomenon attributed to their managerial and leadership roles in health care institutions.

A significant regulatory gap represents the primary structural limitation. Despite widespread adoption driven by practicality and brand reputation, the majority of the 69 identified apps—including the top 13—are not certified as medical devices. This absence of certification extends to highly used resources: evidence databases such as UpToDate and Medscape [51,52], and clinical calculators such as MDCalc and easyPed [53,54]. Although these apps explicitly disclaim medical device status, they routinely perform clinical calculations or influence clinical decision-making, implying that they should be mandated to obtain certification under the EU MDR framework.

Other tools, including ESC CVD Risk Calculator, Cardio4all, MGFamiliar, Elegibilidade THM, and the generative AI tool ChatGPT [55-59], similarly lack formal regulatory status. Poupe Na Receita is the official INFARMED app for medication price consultation, featuring no medical functionalities [60]. On the other hand, national governmental apps like PEM Móvel [61] and SNS 24 [62] operate outside the medical device framework [46,47], relying instead on specific national legislation or informational designations.

Beyond the general use of standard digital tools, our study identified a pioneering niche of early adopters who are already exploring highly advanced technological functionalities. In addition, 3.3% (4/120) of participants reported other specific purposes, namely electronic prescribing and the use of large language models for medical purposes (eg, BioBERT, Med-PaLM, and Med-PaLM 2).

This phenomenon of repurposing apps not classified as mHealth tools, let alone certified as medical devices, has precedent in international literature. Notably, the study by Liu et al [23] in China revealed that physicians rely heavily on nonmedical tools—such as social media and web browsers, used daily by 67% and 34% of respondents, respectively—to support their daily clinical practice, thereby compensating for the lack of specialized medical software tailored to their real-world consultation needs. Consequently, most digital tools in Portuguese GFM practice rely on user trust rather than on formal safety and effectiveness validation, raising concerns about their trustworthiness for clinical decision-making.

Tonic is a notable exception and serves as an international benchmark. As the most widely used app, Tonic (55.8%) demonstrates rigorous certification, including ISO 13485 for medical software, ISO/IEC 42001 for AI management, and UEMS-EACCME accreditation, along with specific modules (eg, calculators and scales) that are registered as medical devices with INFARMED [63-65]. This suggests that validated quality and formal certification promote adherence.

Regarding the MySNS Comunidade digital library, managed by the Shared Services of the Ministry of Health of Portugal (SPMS) and established through normative circular number 02/2018/SPMS [66], it represented an initial attempt to create a national assessment framework. Under this system, the formal evaluation process was designed to be proactively initiated by the app manufacturers themselves. However, this platform is currently no longer available, and the initiative has not been sustained.

Consequently, in Portugal, the only mHealth apps officially recommended and curated by the SNS and the SPMS are almost exclusively those developed by the SPMS itself for the public health system (eg, SNS 24 and PEM Móvel). This creates a potential gap, as physicians may use apps that do not belong to the SPMS and SNS systems. Nevertheless, the initial establishment of this framework was a significant and necessary step. There is an urgent need for national guidelines, as emphasized by 78.4% (104/139) of respondents. In line with a study by Byambasuren et al [22] in Australia, in which 59.9% of physicians identified a lack of knowledge about mHealth apps as the main barrier to their adoption and 15.5% cited a lack of trust in the sources used to access them, physicians expressed the need for a list of mHealth apps—including those on the RACGP’s own list—that are safe and effective in order to overcome these barriers. They also highlighted the need for training guidelines, via online videos or webinars, to learn how to use these technological tools correctly and safely.

Despite the EU MDR 2017/745 operating in Portugal for apps with explicit clinical functions, the vast majority of clinical decision-support systems identified in this study (eg, UpToDate, MDCalc, and Medscape) do not hold formal medical certification, operating within a functional regulatory vacuum and frequently relying on liability disclaimers. Although international models such as Germany’s DiGA specifically evaluate prescribable digital therapeutics for patient use [29] and England’s DTAC establishes broader security benchmarks [19,30], Portugal lacks a centralized national framework to evaluate and curate the digital tools deployed daily within primary care workflows.

Structural barriers such as limited integration with clinical systems (eg, SClínico or SAM) also hinder full adoption of web-based apps and justify the reliance on mobile apps reported by our study participants. Consistent with Zakerabasali et al [67], who highlight barriers such as the lack of structured training and regulatory guidelines, structural barriers remain a key obstacle. Specifically, limited integration with clinical systems (eg, SClínico or SAM) continues to hinder full adoption.

Although Matias Mendes et al [28] primarily highlighted patient-centered barriers, such as low digital literacy (87.3%) and lack of hardware access (81.8%), our research expands this scope by identifying critical structural limitations. We posit that the lack of rigorous scientific validation and official regulation acts as a significant deterrent to prescription.

Consequently, this limits the potential for patients to derive clinical benefit from properly validated digital interventions, underscoring the urgent need for a national evaluation framework to bridge this gap.

Perceptions of AI indicate potential benefits, particularly for clinical decision support and administrative tasks (79/141, 56.0%). The perception and view of AI as a partner for efficiency, freeing up physicians to engage in more face-to-face and relational contact with patients, is highlighted by Martínez-Martínez et al [26]. In Chen et al [24] (China), the overwhelming majority of physicians (75.1%) prefer the term “AI-assisted medicine” to describe the functional role of the technology, emphasizing its supportive nature and ensuring that the physician retains decision-making authority and responsibility.

However, our primary findings reported that AI implementation remains constrained by governance challenges, including algorithmic transparency, data privacy, lack of specific training, and interoperability deficits. In addition, concerns regarding algorithm reliability and the risk of error, expressed by 54.6% (77/141) of respondents, are closely tied to algorithmic “hallucinations” and the inherent lack of transparency within “black box” models [26,41]. Consequently, these challenges primarily stem from concerns regarding the clinical accuracy and safety of the recommendations generated by these digital tools. Additionally, these technical limitations fuel anxieties surrounding algorithmic bias stemming from unrepresentative training datasets, which ultimately threaten to compromise diagnostic equity in primary care [26].

Furthermore, distinct professional barriers exist regarding peer perception. Yang et al [68] found that clinicians who rely on generative AI for decision-making are rated by their peers as having significantly lower clinical skills and competence compared to those who do not use it. Although framing AI usage as a “verification tool” partially mitigates this negative evaluation, it does not fully eliminate the reputational penalty associated with its use. Additionally, the qualitative findings from Martínez-Martínez et al [26] emphasize that AI lacks the ability to recognize and interpret the emotional component characterizing human experience; consequently, it cannot attribute meaning to human distress or demonstrate empathy.

When contrasted with the international literature, our findings highlight a unique adoption paradigm, clarifying this study’s core contribution. Surveys in countries such as Germany and Belgium report significant barriers to mHealth adoption due to strict data privacy and legal liability concerns [20,21], and Australian physicians primarily cite a lack of trusted institutional lists [22]. In contrast, our Portuguese sample exhibits a remarkably high rate of informal adoption (85.1%), although participants admitted the need for official recommendations for apps that have been evaluated for effectiveness and safety.

Similar to findings from China [23], where physicians rely on nonmedical tools to bridge clinical gaps, our study uniquely adds to the literature by demonstrating how “clinical pragmatism”—prioritizing ease of use over formal validation—drives widespread mHealth use in a regulatory vacuum. This underscores a critical global challenge: without accessible, formally evaluated tools (akin to the DiGA or DTAC frameworks) and seamless interoperability, clinicians will independently adopt fragmented, uncertified solutions, risking data security and clinical accuracy.

### Limitations

This study acknowledges several limitations. First, the final sample size (n=141) fell short of the initially calculated target (n=372); although sufficient for descriptive analysis, this resulted in an increased margin of error (8.20%) and reduced statistical power. The lack of representativeness of our sample and the low representation of users and nonusers of apps made comparisons between groups not possible in our study. Second, the online distribution of the survey may have introduced selection bias favoring digitally proactive physicians—generally belonging to a younger age group—and the self-administered format precluded real-time clarification, potentially leading to heterogeneous interpretations. Additionally, this nonprobabilistic convenience sampling limits the statistical generalizability of the findings (external validity); thus, the results should be interpreted as representative of the GFM physicians age groups captured by our sample, rather than the wider population. Nevertheless, this strategy proved indispensable for enabling data collection while ensuring strict confidentiality in instrument access.

Regarding methodological procedures, a primary limitation of this study relates to the sampling and recruitment strategy. The survey was distributed indirectly, relying on intermediary institutions (gatekeepers) to disseminate the link to their contact networks. By delegating the recruitment process to third parties, direct control over the exact size of the population reached was lost. This dispersion precludes the mathematical calculation of conventional response rates (such as viewing or initial participation rates), as the population denominator remains an unknown variable.

Additionally, a minor limitation inherent to web-based surveys is the lack of environmental control over participants’ response behavior. Factors such as leaving the browser tab open during survey completion or rapid clicking could potentially distort the mean completion time. To mitigate this effect and ensure a more accurate representation of the response burden, both the mean and median completion times were analyzed and reported.

Finally, the cross-sectional design limits causal inferences, and the regulatory status assessment was restricted to the 13 most-cited mHealth apps, highlighting the need for broader future evaluation.

### Conclusions

mHealth apps demonstrate a strong trend toward active integration into GFM practice in Portugal among the surveyed cohort. However, as this group was predominantly composed of younger, tech-engaged clinicians, and the final sample size (n=141) remained below the initial target of 372, these findings require further validation on a broader national scale to address selection bias.

To safely scale this adoption, future progress must transition from individual initiative to structured national frameworks that guarantee scientific validation, interoperability with electronic health records (eg, SClínico), and explicit legal safeguards. Ultimately, establishing centralized curation and continuous digital training is essential to prevent health care inequalities and ensure standardized care.

## Supplementary material

10.2196/88760 Multimedia Appendix 1 Survey.

10.2196/88760 Multimedia Appendix 2 Supplementary tables presenting participant characteristics, mobile health app use, perceived benefits, selection and quality criteria, barriers to adoption, and qualitative findings.

## Abbreviations

CHERRIES: Checklist for Reporting Results of Internet E-Surveys
DGS: Directorate-General of Health
DiGA: *Digitale Gesundheitsanwendungen* (Digital Health Applications)
EU: European Union
EURACT: European Academy of Teachers in General Practice/Family Medicine
GDPR: General Data Protection Regulation
GFM: general and family medicine
GP: general practitioner
MDR: Medical Device Regulation
mHealth: mobile health
OM: *Ordem dos Médicos* (Portuguese Medical Association)
RACGP: Royal Australian College of General Practitioners
SNS: *Sistema Nacional de Saúde* (Portuguese National Health Service)
SPMS: *Serviços Partilhados do Ministério da Saúde* (Shared Services of the Ministry of Health of Portugal)
UEMS-EACCME: European Accreditation Council for Continuing Medical Education
WHO: World Health Organization
WONCA: World Organization of Family Doctors
