# Supplementary material for: Use of mHealth Apps by General and Family Medicine Physicians in Portugal: Observational Cross-Sectional Quantitative Study
Source: JMIR Form Res. 2026 Sep 30;10:e88760. doi: 10.2196/88760 (PMC13626549; doi:10.2196/88760)
Supplement: Supplementary file 1 [file formative-v10-e88760-s001.docx]

**Survey**

**Informed Consent**

**“Use of Health Mobile Apps by General and Family Physicians in Portugal”**

*The purpose of this survey is to identify and categorize the health mobile apps currently used in clinical practice within the specialty of General and Family Medicine in Portugal, as well as the criteria that guide clinicians’ decisions regarding the adoption or rejection of these digital tools. Therefore, this survey is directed at general practitioners and family medicine physicians (specialists or residents in the specialty) who are currently practicing in Portugal. Participation in the survey is voluntary and takes approximately 8 minutes to complete.*

*Data are collected anonymously and confidentially and will be used solely for research purposes in accordance with Portugal’s Data Protection Law (Law No. 58/2019 of August 8), which implements, within the national legal framework, the European General Data Protection Regulation (EU 2016/679) and will be stored for a period of 5 years. Participants may request the study results from the researchers. The survey was submitted for review and approval to the Ethics Committee of the Faculty of Medicine at the University of Porto. There are no conflicts of interest.*

*The study was conducted as part of the master’s thesis “Use of Mobile Health Apps in General and Family Medicine Practice in Portugal” by student/researcher Érica Taveira of the University of Porto to earn a master’s degree in Medical Informatics under the supervision of Prof. Inês Dutra, in collaboration with Fraunhofer Portugal AICOS under the supervision of researcher Sílvia Rêgo.*

***Érica Taveira (up202308132@up.pt)***

***Inês Dutra (dutra@fc.up.pt)***

***Sílvia Rêgo (silvia.rego@aicos.fraunhofer.pt)***

***Indicates a required question**

**Q1. (Single-choice question) ***

I declare that I have read and understood the introductory explanation about this survey and how the collected data will be used, and I hereby consent to participate in this study. *

• Yes (Redirected to the “Section 1-Practice of General and Family Medicine (GFM)” section)

• No (Redirected to the end of the survey)

**Section 1-Practice of General and Family Medicine (GFM) (Q2-Q3;2 items)**

**Q2. (Single-choice question) ***

Are you a General and Family Medicine (GFM) physician registered with the Portuguese Medical Association, or are you currently completing your residency in GFM in Portugal?

• Yes.

• No. (Redirected to the “End of Survey” section)

**Q3. (Single-choice question) ***

Are you currently practicing General and Family Medicine (GFM) in Portugal in the public, private, or social sector?

• Yes. (Redirected to “Section 2-Demographic Data on Physicians Specializing in General and Family Medicine (MGF)”)

• No. (Redirected to the “End of Survey” section)

**Section 2-Demographic Data on Physicians Specializing in General and Family Medicine (GFM) (Q4-Q9; 6 items)**

**Q4. (Single-choice question) ***

**Select the option that corresponds to your age group.**

• Under 26 years old

• 26 to 35 years old

• 36 to 45 years old

• 46 to 55 years old

• 56 to 65 years old

• 66 to 75 years old

• 76 to 85 years old

• Over 85 years old

**Q5. (Single-choice question) ***

**Select the gender with which you identify.**

• Female

• Male

• Other

• I’d rather not say

**Q6. (Single-choice question) ***

**Select the role you currently hold.**

• General and Family Medicine Specialist

• Resident specializing in General and Family Medicine

**Q7. (Single-choice question) ***

Select the option corresponding to the number of years you have been practicing General and Family Medicine after completing your core residency year (excluding periods of interruption or transition).

• < 5 years

• 5 to 15 years

• 16 to 25 years

• 26 to 35 years

• 36 to 45 years

• ≥ 46 years

**Sector(s) in which you practice family medicine**

**Q8. (Multiple-choice question) ***

In which sector(s) do you practice family medicine? (Select all applicable sectors)

• Public

• Private

• Social

**Q9. (Multiple-choice question) ***

Select the district(s) or autonomous region(s) in which you practice General Family Medicine.

• Azores

• Aveiro

• Beja

• Braga

• Bragança

**•** Castelo Branco

• Coimbra

• Évora

• Faro

• Guarda

• Leiria

• Lisbon

• Madeira

• Portalegre

• Porto

• Santarém

• Setúbal

• Viana do Castelo

• Vila Real

• Viseu

**(Refer to “Section 3-Use of Mobile Health Apps in the Clinical Practice of GFM”)**

**Section 3-Use of Mobile Health Apps in the Clinical Practice of GFM (Q10-Q15;4-5 items)**

**Q10. (Single-choice question) ***

Are you aware that there are mobile health apps designed for the clinical practice of GFM?

• Yes

• No

**Q11. (Single-choice question) ***

Do you use mobile health apps in your clinical practice related to GFM?

• Yes (Proceed to question “Q12.”)

• No (Proceed to question “Q14.”)

**Q12. (Multiple-choice question) ***

**What benefits do you see in using mobile health apps in your clinical practice regarding GFM?**

(Select all that apply)

• Access to clinical evidence to support clinical practice

• Real-time availability of patients’ health data

• Insight into and feedback on patients’ adherence to prescribed treatment

• Availability of a digital history of updates to patients’ electronic health records

• Automatic alerts for drug interactions and contraindications

• Digital checklists or guidelines for standardized procedures and diagnoses

• Automation of administrative tasks

• Workflow management systems

• Simplified data entry with voice or touch recognition features

• Automatic data integration into centralized health systems

• Teleconsultations and remote monitoring

• Organization of clinical tasks with automated alerts and reminders

• Efficient scheduling of appointments and procedures

• I do not believe there are any benefits

• Other options

**Q13. (Open-ended question)**

**If you previously selected the option “Other options,” please briefly and objectively describe the benefits you see in the use of mobile health apps in the context of GFM.**

**(This question pertains to “Section 4-Identification, Categorization, and Characterization of Mobile Health Apps in the Context of GFM.”)**

**Q14. (Multiple-choice question) ***

**If you were to use mobile health apps in your clinical practice, what benefits would you identify or would they bring you? (Select all that apply)**

• Access to clinical evidence to support clinical practice

• Real-time availability of patients’ health data

• Insight into and feedback on patients’ adherence to prescribed treatment

• A digital history of updates to patients’ electronic health records

• Automatic alerts for drug interactions and contraindications

• Digital checklists or guidelines for standardized procedures and diagnoses

• Automation of administrative tasks

• Workflow management systems

• Simplified data entry with voice or touch recognition features

• Automatic data integration into centralized health systems

• Teleconsultations and remote monitoring

• Organization of clinical tasks with automated alerts and reminders

• Efficient scheduling of appointments and procedures

• I do not believe there are any benefits

• Other options

**Q15. (Open-ended question)**

**If you previously selected the option “Other options,” please briefly and objectively describe the benefits you see in the use of mobile health apps in the context of GFM.**

**(This question pertains to “Section 5-Knowledge regarding the use of mobile health apps in the field of GFM in Portugal.”)**

**Section 4-Identification and Categorization of Health Apps in the Context of GFM (O16-Q18; 2-3 items)**

*We recommend that you check the health apps installed on your mobile device (SPMS apps, utilities, exam tools, and others) before answering the questions in this section.*

**Q16. (Multiple-choice question) ***

**Indicate the purpose(s) for which you use mobile health apps (Select all that apply):**

• Prescribing mobile health apps as part of patients’ treatment plans

• Prescribing health mobile apps to patients for tracking and monitoring their medical conditions

• Supporting clinical decision-making (e.g., consulting digital checklists or guidelines for standardized procedures and diagnoses)

• Searching for scientific evidence to support clinical practice

• Patient health education and awareness

• Using mobile health apps to communicate with patients (messages, teleconsultations, etc.)

• Management of appointments, scheduling, and access to patients’ clinical records

• Management and monitoring of patients’ medication adherence

• Use of calculators and clinical assessment tools (e.g., risk scores)

• Sharing patients’ health data with other healthcare professionals as part of workflows in use at your workplace

• Sharing (anonymized) clinical cases among healthcare professionals to obtain clinical opinions on the cases in question

• Other purpose(s)

**Q17. (Open-ended question)**

If you previously selected the option “Other purpose(s),” please specify.

**Q18. (Single-choice question) ***

How many health mobile apps do you use in the practice of GFM or recommend to your patients?

• 1 (Redirects to “Section 4.1 – Option selected: Use of 1 health mobile app”)

• 2–5 (Redirected to “Section 4.2 – Option selected: Use of 2–5 health mobile apps”)

• 6–10 (Redirected to “Section 4.3 – Option selected: Use of more than 5 health mobile apps”)

**Sections 4.1, 4.2 e 4.3**

**Note: The display of the initial questions in these sections is conditional, adapting dynamically to the option selected in the initial filtering question: - Selecting '1' redirects to 'Section 4.1 (Q19–Q36; 9–18 items)—Option selected: Use of 1 mobile health app' (displayed once). - Selecting '2–5' redirects to 'Section 4.2 (Q37–Q74; 12–38 items )—Selected option: Use of 2–5 mobile health apps' (allowing respondents to provide information for a maximum of 5 apps). - Selecting '6–10' redirects to 'Section 4.3 (Q75–Q137; 12–63 items)—Selected option: Use of more than 5 mobile health apps' (allowing respondents to provide information for a maximum of 10 apps).**

**Q19. (Open-ended question) ***

Name of the health mobile app (If you don’t remember, enter a 0):

**Q20. (Multiple-choice question) ***

Category of the health mobile app:

• Management of Chronic Diseases and Conditions

• Well-being and Lifestyle

• Health Education

• Monitoring of Physiological Parameters

• Medication Management

• Telemedicine and Communication with Healthcare Professionals

• Clinical Practice Support

• Mental Health

• Pregnancy and Reproductive Health

• Public Health and Prevention

• Rehabilitation and Physical Therapy

• Sexual Health

• Medical Emergency Support

• Nutrition and Diet Management

• Other option

**Q21. (Open-ended question)**

If you previously selected the “Other option” choice, please specify the category for health mobile app 1.

Q22. (Multiple-choice question) *

List the main features or reasons why you use the health mobile app (Select all that apply):

• Management of chronic diseases and conditions (e.g., diabetes, hypertension)

• Promoting well-being and a healthy lifestyle (e.g., physical activity tracking, meditation)

• Health education (e.g., access to educational content, information about medical conditions)

• Monitoring physiological parameters (e.g., heart rate, blood glucose)

• Medication management (e.g., medication reminders)

• Telemedicine and communication with healthcare professionals (e.g., remote consultations, secure messaging)

• Clinical practice support (e.g., calculation of indices such as BMI, clinical decision algorithms)

• Mental health (e.g., mood monitoring, digital therapy)

• Pregnancy and reproductive health (e.g., prenatal care, fertility calendars)

• Public health and prevention (e.g., screenings, vaccination campaigns)

• Rehabilitation and physical therapy (e.g., guided exercises, progress tracking)

• Sexual health (e.g., STI screening, sex education)

• Medical emergency support (e.g., basic and advanced life support algorithms)

• Nutrition and diet management (e.g., calorie tracking, meal planning)

• Other(s) (specify)

**Q23. (Open-ended question)**

If you previously selected the “Other” option, please specify the features or reasons for using the mobile health app.

**This question structure appears only once and is mandatory within Sections 4.1, 4.2, and 4.3.**

**Q24. (Single-choice question) ***

Select the option that most closely matches your actual frequency of use of health mobile app 1 over the past year (using today as a reference):

• Daily

• Weekly

• Monthly

• Quarterly

• Semiannually

• Annually

• Other

**Q25. (Open-ended question)**

If you previously selected “Other,” please specify how often you use health app 1.

**Q26. (Single-choice question) ***

Indicate the primary operating system of the mobile device you use in your clinical practice:

• Android®

• iOS®

• Other option

**Q27. (Open-ended question)**

If you previously selected the “Other option” choice, please specify the primary operating system of the mobile device you use in your clinical practice.

**Q28. (Multiple-choice question) ***

In what contexts do you typically use mobile health apps in your clinical practice of GFM? (Select all that apply)

• During a medical consultation (e.g., diagnostic support, access to guidelines)

• Outside of consultations, for planning or reviewing clinical cases (e.g., schedule management, treatment reviews)

• When communicating with other healthcare professionals (e.g., discussing clinical cases)

• In the remote monitoring of patients (e.g., telemonitoring, receiving data sent by mobile health apps)

• In direct patient counseling (e.g., demonstrating features, digital prescribing)

• For continuing education and training (e.g., reading scientific articles, participating in webinars, learning new protocols)

• In urgent or emergency situations (e.g., support algorithms, rapid protocols)

• Other context(s) (please specify)

**Q29. (Open-ended question)**

If you previously selected the option “Other context(s),” please specify.

**Q30. (Multiple-choice question) ***

Regarding your use of the health mobile apps you indicated earlier, this was due to:

• Guidance from the Directorate-General for Health (DGS)

• Guidance from the World Health Organization (WHO)

• Guidance from the institution where you practice

• Recommendation from the DGS

• Recommendation from the WHO

• Recommendation from the institution where you practice

• Recommendation from the General Practice and Family Medicine Specialty Board

• Recommendation from the Portuguese Association of General and Family Medicine

• Recommendation from another association, society, federation, council (national or international), or other body in a specialty other than General and Family Medicine

• Recommendation from one or more colleagues

• On one’s own initiative

**Q31. (Multiple-choice question)**

If you previously selected “Yes” for the option “Recommendation from another association (national or international) in a specialty other than FGM,” please specify the medical specialties other than FGM.

• Pathology

• Anesthesiology

• Angiology and Vascular Surgery

• Cardiology

• Pediatric Cardiology

• Cardiothoracic Surgery

• General Surgery

• Oral and Maxillofacial Surgery

• Pediatric Surgery

• Plastic, Reconstructive, and Aesthetic Surgery

• Dermatology and Venereology

• Infectious Diseases

• Endocrinology and Nutrition

• Stomatology

• Clinical Pharmacology

• Gastroenterology

• Medical Genetics

• Gynecology/Obstetrics

• Clinical Hematology

• Immunoallergology

• Immunohemotherapy

• Sports Medicine

• Occupational Medicine

• Physical Medicine and Rehabilitation

• Intensive Care Medicine

• Internal Medicine

• Forensic Medicine

• Nuclear Medicine

• Tropical Medicine

• Nephrology

• Neurosurgery

• Neurology

• Neuroradiology

• Ophthalmology

• Medical Oncology

• Orthopedics

• Otolaryngology

• Clinical Pathology

• Pediatrics

• Pulmonology

• Psychiatry

• Child and Adolescent Psychiatry

• Radiology

• Radiation Oncology

• Rheumatology

• Public Health

• Urology

• Other specialty(ies)

**Q32. (Open-ended question)**

If you previously selected the option “Other specialty(ies),” please specify.

**Q33. (Multiple-choice question) ***

What are the decision-making criteria that guide your use of the aforementioned mobile health app in your clinical practice of general family medicine?

• Proven clinical efficacy/effectiveness

• Ease of use

• Data security and privacy

• Cost of the app

• Recommendations from institutions or colleagues

• Compatibility with existing systems (e.g., integration with clinical software in use, such as hospital management systems or health databases)

• User acceptance

• Availability on different platforms (iOS, Android)

• Updates and technical support

• Customization to specific clinical needs

• Other(s)

**Q34. (Open-ended question)**

If you previously selected the “Other(s)” option, please specify the decision criteria for using the mobile health app(s).

**Q35. (Multiple-choice question) ***

What are the quality criteria that guide your use of the mobile health app in your clinical practice of GFM? (Select all that apply)

• Efficacy validated by scientific studies

• Intuitive and accessible interface

• Compliance with privacy regulations (e.g., GDPR)

• Clinical relevance to FGM practice

• Integration with existing digital systems

• Robust cybersecurity safeguards

• Credibility of the developer or responsible institution

• Regular updates with improvements based on scientific evidence

• Efficient and accessible technical support

• Customization to specific clinical needs

• Proven acceptance by users and professionals

• Other(s)

**Q36. (Open-ended question)**

If you previously selected the option “Other(s),” please specify the quality criteria for the use of mobile health applications.

**(Redirected to *“Section 6 — Opinions and perspectives on the use of currently available mobile health apps”*)**

**Section 5—Knowledge Regarding the Use of Mobile Health Apps in Family Medicine in Portugal** (**Q138–Q170; 5–33 items).**

**Q138. (Multiple-choice question) ***

Please indicate the reason or reasons why you do not use mobile health apps in your clinical practice.

• Lack of knowledge or familiarity with available mobile health apps

• Concerns about the security and privacy of health data

• Lack of integration with existing clinical systems

• Perception that mobile health apps do not contribute significantly to patient care

• Insufficient time to explore or use new tools

• Preference for traditional methods of scheduling appointments

• Preference for traditional methods of patient follow-up and monitoring

• Lack of training on the subject

• Lack of adequate technical support

• Costs associated with purchasing or maintaining the apps

• Difficulties patients have adapting to digital tools

• Other option

**Q139. (Open-ended question)**

If you previously selected the “Other option” choice, please specify the reason(s) for not using mobile health apps.

**Q140. (Single-choice question)**

**Are you aware of any health mobile apps used in the field of OB/GYN in Portugal?**

- Yes
- No

**Intermediate section**

Please list the names and categories of the health mobile apps (up to 10 health mobile apps) that you are familiar with.

**For the sake of brevity in this document, the repeating question structure is displayed as a single-application template; however, the electronic survey functionally allows respondents to input identical information for up to 10 distinct applications.**

**Q141. (Open-ended question) ***

Name of health mobile app 1 (If you cannot recall, enter a 0):

**Q142. (Multiple-choice question) ***

Category of health mobile app 1:

• Management of Chronic Diseases and Conditions

• Well-being and Lifestyle

• Health Education

• Monitoring of Physiological Parameters

• Medication Management

• Telemedicine and Communication with Healthcare Professionals

• Clinical Practice Support

• Mental Health

• Pregnancy and Reproductive Health

• Public Health and Prevention

• Rehabilitation and Physical Therapy

• Sexual Health

• Medical Emergency Support

• Nutrition and Diet Management

• Other

**Q143. (Open-ended question)**

If you previously selected “Other,” please specify the category of mobile app 1.

**(Redirected to the section *“Section 6—Opinions and Perspectives on the Use of Currently Available Mobile Health Apps*”)**

**Section 6—Opinions and Perspectives on the Use of Currently Available Mobile Health Apps (Q171-Q182; 10-12 items)**

**Q171. (Likert scale question) ***

To what extent do you consider the available scientific evidence sufficient to validate the use of mobile health apps in the clinical practice of GFM?

• Level of satisfaction: Insufficient, Somewhat sufficient, Sufficient, Very sufficient

**Q172. (Likert scale question) ***

Do you trust the quality and safety of the information provided by currently available mobile health apps?

• Level of confidence: None, Very low, Low, Moderate, High, Very high

**Q173. (Single-choice question) ***

Do you believe there is sufficient outreach and training provided by the DGS for healthcare professionals regarding health mobile apps for use in the clinical practice of GFM?

• Yes

• No

**Q174. (Single-choice question) ***

Would you like to receive more specific training on the use of GFM health mobile apps?

• Yes

• No

**Q175. (Likert scale question) ***

Do you agree that knowledge—or lack thereof—regarding health mobile apps designed to support FGM clinical practice influences their use in your clinical practice?

• Level of agreement: Strongly disagree, Disagree, Neither agree nor disagree, Agree, Strongly agree

**Q176. (Likert scale question) ***

In your opinion, to what extent do you agree that the recommendations, standards, or guidelines available at the national level regarding mobile health apps are sufficient to support FGM physicians in their decision to use them in clinical practice?

• Level of agreement: Strongly disagree, Disagree, Neither agree nor disagree, Agree, Strongly agree

**Q177. (Multiple-choice question) ***

What do you consider to be the biggest barriers to the adoption of mobile health apps in your clinical practice?

• Lack of scientific evidence

• Lack of awareness of existing mobile health apps

• Health data privacy concerns

• Resistance from patients

• Lack of specific guidelines from the National Health Service

• Lack of specific guidelines from national scientific bodies (e.g., scientific societies, specialty colleges, etc.)

• Lack of specific guidelines from international scientific bodies (e.g., WHO, international scientific societies, etc.)

• I do not believe there are any barriers

• Other option

**Q178. (Open-ended question)**

If you previously selected the “Other option” choice, please specify.

**Q179. (Single-choice question) ***

Do you consider it necessary for the National Health System (SNS) to issue specific guidelines on the use of health mobile apps in the context of FGM?

• Yes

• No

**Q180. (Multiple-choice question) ***

What kind of support or regulation do you expect from the National Health System (SNS) regarding the use of health mobile apps?

(Select all options you consider applicable)

• Clear guidelines for use

• Scientific evaluation of health mobile apps

• Evaluation of health mobile apps regarding user data security and protection

• Information on which health mobile apps are available

• Continuing education

• Other option

**Q181.(Open-ended question)**

If you previously selected the “Other option” choice, please specify.

**Q182. (Likert scale question) ***

Do you believe that mobile health apps should be integrated into the National Health Service (SNS) to support clinical practice?

**•** Level of agreement: Strongly disagree, Disagree, Neither agree nor disagree, Agree, Strongly agree

(Directed to the section “*Section 7—Opinions and perspectives on the use of Artificial Intelligence (AI) integrated into mobile health apps in primary care practice*”)

**Section 7—Opinions and Perspectives on the Use of Artificial Intelligence (AI) Integrated into Mobile Health Apps in the Clinical Practice of GFM** (**Q183-Q193; 5-11 items)**

**Q183. (Likert scale question) ***

Are you aware of any mobile health apps for the clinical practice of FGM that integrate Artificial Intelligence (AI)?

• Yes

• No

**Q184. (Open-ended question)**

If you can recall, please list the names of the mobile health apps you are aware of for clinical GFM practice that integrate AI.

**Q185. (Multiple-choice question) ***

Do you believe that AI tools integrated into mobile health apps can be beneficial in improving clinical GFM practice?

• Yes

• No

**Q186. (Multiple-choice question)**

If you previously answered “Yes,” please specify the areas of clinical FGM practice that could benefit from the use of mobile health apps integrated with AI tools. (Select all options you consider applicable)

• Clinical decision support

• AI-assisted diagnosis

• Remote patient monitoring

• Clinical data management

• Automation of administrative tasks

• Telemedicine and patient triage

• Medical education and continuing education

• Other(s)

**Q187. (Open-ended question)**

If you previously selected the option “Other(s),” please specify.

**Q188. (Multiple-choice question) ***

What are the main challenges you identify in the implementation and use of Artificial Intelligence (AI) in the clinical practice of general medicine? (Select all options you consider applicable)

• Lack of specific training in AI

• Ethical, security, and health data privacy issues

• Financial costs of implementation

• Resistance among healthcare professionals to adopting the technology

• Integration and interoperability with currently used systems (e.g., SClínico, PEM, etc.)

• Reliability of algorithms and risk of clinical errors

• I do not identify any challenges

• Other(s)

Q189. (Open-ended question)

If you previously selected the “Other(s)” option, please specify.

**Q190. (Multiple-choice question) ***

*This question is intended only for respondents who use mobile health apps in the clinical practice of FGM; if this does not apply to you, please proceed to the next question. *

Do you know whether the mobile health apps you use in the clinical practice of GFM incorporate AI tools?

• Yes, they incorporate AI tools.

• No, they do not incorporate AI tools.

• I do not know.

**Q191. (Multiple-choice question) ***

How do you assess the impact of using AI in the clinical practice of GFM on the doctor-patient relationship?

• It improves the relationship, allowing more time for the patient

• It does not significantly affect the relationship

• It harms the relationship, making it more impersonal (less humanized)

• I have not formed an opinion on the subject

• Other(s)

**Q192. (Open-ended question)**

If you previously selected the “Other(s)” option, please specify the impacts you consider relevant.

**Q193. (Open-ended question)**

If you consider it relevant, please leave a comment.

(Directed to the *“End of the survey”* section)

**Survey Completion**

*Dear participant,*

*Please be advised that the survey has been closed, as the objective of this study is limited to the current practice of General and Family Medicine. Thank you for your interest and willingness to participate. If you would like to receive the results of this study, please complete the following survey (1 minute) available at (insert Google Forms® link). If you have any questions, please do not hesitate to contact us via email at up202308132@up.pt (Érica Taveira), silvia.rego@aicos.fraunhofer.pt (Sílvia Rêgo), or dutra@fc.up.pt (Inês Dutra).*

*Sincerely,*

*Érica Taveira*

**End of survey**

*Dear participant,*

*Please be advised that the survey has ended. Thank you for your interest and willingness to participate. If you would like to receive the results of this study, please complete the following survey (1 minute) available at (insert Google Forms® link) If you have any questions, please do not hesitate to contact us via email at up202308132@up.pt, silvia.rego@aicos.fraunhofer.pt, or dutra@fc.up.pt.*

*Sincerely,*

*Érica Taveira*

**Survey for Submitting Study Results**

**Submission of Study Results - “Use of Mobile Health Apps in General and Family Medicine Clinical Practice in Portugal”**

*This survey is intended for submitting the results of the study “Use of mobile health applications in the clinical practice of general and family medicine in Portugal” by student/researcher Érica Taveira of the University of Porto for her master’s degree in Medical Informatics under the supervision of Prof. Inês Dutra, in collaboration with Fraunhofer Portugal AICOS under the supervision of researcher Sílvia Rêgo.*

*The data are collected anonymously and confidentially and are intended to be used solely for research purposes in accordance with the Portuguese Data Protection Law (Law No. 58/2019 of August 8), which implements, within the national legal framework, the European General Data Protection Regulation (EU 2016/679) into national law, and will be stored for a period of 5 years. Participants may request the study results from the researchers. The survey was submitted for review and approval to the Ethics Committee of the Faculty of Medicine at the University of Porto. There is no conflict of interest.*

***Érica Taveira (up202308132@up.pt)***

***Inês Dutra (dutra@fc.up.pt)***

***Sílvia Rêgo (silvia.rego@aicos.fraunhofer.pt)***

**Q1. (Open-ended question)**

**Please provide the email address where you would like to receive the results of this study.**

*Dear participant,*

*Please be advised that the survey for sending the results of the study “Use of Mobile Health Apps in General and Family Medicine Clinical Practice in Portugal” has ended. Thank you for your interest and participation. If you have any questions, please do not hesitate to contact us via email at up202308132@up.pt (Érica Taveira), silvia.rego@aicos.fraunhofer.pt (Sílvia Rêgo), or dutra@fc.up.pt (Inês Dutra).*

*Sincerely,*

*Érica Taveira*

**End of survey**
