# Supplementary material for: Use of mHealth Apps by General and Family Medicine Physicians in Portugal: Observational Cross-Sectional Quantitative Study
Source: JMIR Form Res. 2026 Sep 30;10:e88760. doi: 10.2196/88760 (PMC13626549; doi:10.2196/88760)
Supplement: Supplementary file 2 [file formative-v10-e88760-s002.docx]

Multimedia Appendix 2: Supplementary Tables (Tables S1-S9)

Table S1. Districts and autonomous regions where participants practice General and Family Medicine (n=141)

| **Districts and autonomous regions where participants practice General and Family Medicine, n (%)** |  |  |
| --- | --- | --- |
|  | Aveiro | 21 (14.9) |
|  | Braga | 12 (8.5) |
|  | Braga and Lisboa | 1 (0.7) |
|  | Braga and Porto | 3 (2.1) |
|  | Bragança | 2 (1.4) |
|  | Castelo Branco | 1 (0.7) |
|  | Coimbra | 1 (0.7) |
|  | Évora | 1 (0.7) |
|  | Faro | 5 (3.5) |
|  | Guarda | 1 (0.7) |
|  | Leiria | 1 (0.7) |
|  | Lisboa | 33 (23.4) |
|  | Madeira | 1 (0.7) |
|  | Portalegre | 2 (1.4) |
|  | Porto | 38 (27.0) |
|  | Santarém | 3 (2.1) |
|  | Setúbal | 9 (6.4) |
|  | Vila Real | 2 (1.4) |
|  | Vila Real and Viseu | 1 (0.7) |
|  | Viseu | 3 (2.1) |

Table S2. Perceived benefits identified by mHealth app users (n=120) and non-users as hypothetical users (n=21)

| **Benefits, n(%)** |  | **Users (n=120)** | **Non-users (n=21)** |
| --- | --- | --- | --- |
|  | Access to clinical evidence to support clinical practice | 115 (95.8) | 15 (71.4) |
|  | Real-time availability of patient health data | 24 (20.0) | 10 (47.6) |
|  | Insights and feedback on patients’ adherence to prescribed treatment | 21 (17.5) | 8 (38.1) |
|  | Availability of a digital history of updates to patients’ electronic health records | 30 (25.0) | 9 (42.9) |
|  | Automatic alerts for drug interactions and contraindications | 43 (35.8) | 14 (66.7) |
|  | Digital checklists or guidelines for standardized procedures and diagnoses | 89 (74.2) | 10 (47.6) |
|  | Automation of administrative tasks | 29 (24.2) | 8 (38.1) |
|  | Workflow management systems | 19 (15.8) | 4 (19.0) |
|  | Simplified data entry with voice or touch recognition features | 13 (10.8) | 5 (23.8) |
|  | Automatic data integration into centralized healthcare systems | 27 (22.5) | 9 (42.9) |
|  | Teleconsultations and remote monitoring | 18 (15.0) | 6 (28.6) |
|  | Organization of clinical tasks with automated alerts and reminders | 28 (23.3) | 8 (38.1) |
|  | Efficient scheduling of appointments and procedures | 18 (15.0) | 7 (33.3) |
|  | I do not believe there is any benefit | 0 (0.0) | 1 (4.8) |
|  | Other options | 4 (3.3) | 1 (4.8) |

## Table S3. mHealth apps identified by the participants (n=120)

| **Name of mHealth app, n (%)** |  |  |
| --- | --- | --- |
|  | ACCESS | 1 (0.8) |
|  | ADS | 1 (0.8) |
|  | ALERT | 1 (0.8) |
|  | AlgoritMD | 3 (2.5) |
|  | AMBOSS | 1 (0.8) |
|  | AnthroCalc | 1 (0.8) |
|  | AnticoagEvaluator (Am Coll Card) | 1 (0.8) |
|  | APMGF MOBILE | 1 (0.8) |
|  | Calculate by QxMD | 3 (2.5) |
|  | Cardio4all | 26 (21.7) |
|  | ChatGPT | 4 (3.3) |
|  | Clinical Frailty Scale (CFS) | 1 (0.8) |
|  | ClinicoMais | 2 (1.7) |
|  | Clue | 1 (0.8) |
|  | Contraceção.pt | 1 (0.8) |
|  | Contraception | 1 (0.8) |
|  | Cronometer | 1 (0.8) |
|  | Diabetes:M | 1 (0.8) |
|  | Dioscope | 1 (0.8) |
|  | Dor.com.pt | 1 (0.8) |
|  | Dr.Share | 2 (1.7) |
|  | Dynamed | 3 (2.5) |
|  | easyPed | 22 (18.3) |
|  | Elegibilidade THM | 4 (3.3) |
|  | ePed | 2 (1.7) |
|  | ESC CVD Risck Calculator | 9 (7.5) |
|  | ESC Guidelines | 1 (0.8) |
|  | ESC Pocket Guidelines | 1 (0.8) |
|  | fatsecret | 1 (0.8) |
|  | febre-i-dor | 1 (0.8) |
|  | Fit Google | 1 (0.8) |
|  | Flo.Health App | 1 (0.8) |
|  | FRAX Portugal | 2 (1.7) |
|  | Google Lens | 1 (0.8) |
|  | Guias SPMI | 1 (0.8) |
|  | Hábil | 1 (0.8) |
|  | IAM Medical Guidelines 2.0 | 1 (0.8) |
|  | iMed | 1 (0.8) |
|  | Infomed | 1 (0.8) |
|  | Libreview | 1 (0.8) |
|  | Manual MSD | 1 (0.8) |
|  | MDCalc | 25 (20.8) |
|  | MediCalc | 2 (1.7) |
|  | Médis Active | 1 (0.8) |
|  | Med-Palm | 1 (0.8) |
|  | Medscape | 13 (10.8) |
|  | MGFamiliar | 4 (3.3) |
|  | Migraine buddy | 1 (0.8) |
|  | My Santa Maria | 1 (0.8) |
|  | OB Wheel | 2 (2.7) |
|  | Open WHO | 1 (0.8) |
|  | OpenEvidence | 3 (2.5) |
|  | Outlook + Office 365 no global | 1 (0.8) |
|  | PEM Móvel | 26 (21.7) |
|  | Poupe na Receita | 4 (3.3) |
|  | Pregnancy Calculator | 1 (0.8) |
|  | ReumaGuide | 2 (1.7) |
|  | RheumaHelper | 1 (0.8) |
|  | RSE- live | 2 (1.7) |
|  | Simposium | 1 (0.8) |
|  | Snore Lab | 1 (0.8) |
|  | SNS 24 | 8 (6.7) |
|  | Tät Pelvic floor exercises | 1 (0.8) |
|  | T-Lab | 1 (0.8) |
|  | Tonic | 67 (55.8) |
|  | UpHill | 1 (0.8) |
|  | UpToDate | 34 (28.3) |
|  | USPSTF | 1 (0.8) |
|  | White Noise Generation | 1 (0.8) |

Table S4. Criteria for mHealth apps (n=120)

| **Criteria, n(%)** |  |  |
| --- | --- | --- |
|  | Ease of use | 114 (95.0) |
|  | Clinical efficacy proven | 79 (65.8) |
|  | Cost of the app | 64 (53.3) |
|  | Availability on different platforms (e.g., iOS, Android) | 38 (31.7) |
|  | Recommendations by colleagues or institutions | 38 (31.7) |
|  | Updates and technical support | 32 (26.7) |
|  | Customization to meet specific clinical needs | 32 (26.7) |
|  | Data security and privacy | 27 (22.5) |
|  | Compatibility with existing systems | 17 (14.2) |
|  | User acceptance | 10 (8.3) |

Table S5. Quality criteria for the selection of mHealth apps (n=120)

| **Quality criteria, n (%)** |  |  |
| --- | --- | --- |
|  | Clinical relevance for GFM practice | 102 (85.0) |
|  | Efficacy validated by scientific studies | 77 (64.2) |
|  | Intuitive and accessible interface | 77 (64.2) |
|  | Regular evidence-based updates | 62 (51.7) |
|  | Customization to specific clinical needs | 38 (31.7) |
|  | Credibility of the developer/institution | 37 (30.8) |
|  | Proven acceptance by users and professionals | 27 (22.5) |
|  | Compliance with privacy regulations (GDPR) | 23 (19.2) |
|  | Efficient and accessible technical support | 20 (16.7) |
|  | Integration with existing digital systems | 13 (10.8) |
|  | Guaranteed cybersecurity | 12 (10.0) |
|  | Others | 1 (0.8) |

Table S6. Reasons for the non-use of mHealth apps (n=21)

| **Reasons, n(%)** |  |  |
| --- | --- | --- |
|  | Lack of integration with clinical systems | 15 (71.4) |
|  | Insufficient time to explore/use | 15 (71.4) |
|  | Lack of knowledge/familiarity | 9 (42.9) |
|  | Lack of technical support | 9 (42.9) |
|  | Associated costs | 7 (33.3) |
|  | Lack of training | 7 (33.3) |
|  | Data security/privacy | 5 (23.8) |
|  | Preference for traditional methods (follow-up) | 5 (23.8) |
|  | Difficulties users have adapting | 5 (23.8) |
|  | Preference for traditional methods (scheduling) | 3 (14.3) |
|  | Others | 1 (4.8) |
|  | Perception of no clinical benefit | 0(0.0) |

Table S7. Barriers to adoption of mHealth apps (n=139)

| **Barriers, n(%)** |  |  |
| --- | --- | --- |
|  | Lack of awareness of existing mHealth apps solutions | 117(84.2) |
|  | Lack of specific guidelines from the National Health Service (SNS) | 66(47.5) |
|  | Lack of specific guidelines from the national scientific bodies (e.g., scientific societies, specialty colleges, etc.) | 66(47.5) |
|  | Health data privacy issues | 42(30.2) |
|  | Lack of specific guidelines from international scientific bodies (e.g., WHO, international scientific societies, etc.) | 41(29.5) |
|  | Resistance from patients | 29(20.5) |
|  | Other option | 16(11.5) |
|  | Lack of scientific evidence | 9(6.5) |
|  | I do not believe there are any barriers | 3(2.2) |

Table S8. Qualitative themes and operational barriers identified through open-ended responses

| **Barrier category** | **Specific Technical and Operational Challenges** |
| --- | --- |
| Technological infrastructure limitations | Insufficient internet coverage; inability to install apps on institutional workstations; and National Health Service (SNS) information systems described as ‘terrible’ or outdated. |
| Deficient clinical software integration | Lack of connection to programs used during consultations; burden of managing simultaneous multiple platforms; and a lack of standardization across Local Health Units (ULSs). |
| Organizational and time constraints | Severe time limitations during consultations; difficulties in implementing these tools in patient care; and an overload of digital tasks. |
| Patient and user-related factors | Digital illiteracy and technological limitations among patients, alongside a baseline lack of interest among some physicians in using digital platforms. |
| Economic and regulatory aspects | Cost of applications and the failure to update or make available DGS/WHO guidelines in an accessible, user-friendly format. |

Table S9. Qualitative themes and perspectives of the participants identified through open-ended responses regarding AI

| **Attitudinal Theme/Dimension** | **Illustrative Quotes and Perspectives** |
| --- | --- |
| Transformative potential | *‘AI provides analytical power of superhuman intensity. Putting all that power at the service of our users is a real asset.’* |
| Regulatory imperatives | *‘If properly regulated, it will be an indispensable/very useful tool/partner.’* |
| Systemic skepticism and caution | *‘t’s too early to have a concrete perspective on the applicability of AI. There’s also a lack of legal support/civil liability.’* |
| Prerequisite infrastructural conditions | *‘We’re in a terrible situation when it comes to basic applications like SClínico and PEM; until that’s resolved, there’s no point in adding more steps.’* |
| Proposed clinical applications | Direct support for appointment triage, clinical record-keeping optimization, symptoms and signs search, and moderating demand at primary care centers. |
| Mixed professional feelings | Institutional concern (*‘Many questions, various fears, and high potential’*) |
| Responses range from high enthusiasm and even gratitude | *(‘Excellent research topic!’*) and (*‘Thank you for conducting this study! Keep it up!’*). |
